# Multiscale characterization of lipid bilayer disruption induced by Methyl-β-cyclodextrin (MβCD)

**DOI:** 10.1007/s00249-026-01821-y

**Published:** 2026-01-29

**Authors:** Wisnu Arfian Anditya Sudjarwo, Jose L. Toca-Herrera

**Affiliations:** Department of Natural Sciences and Sustainable Resources, BOKU University, Institute of Biophysics, Muthgasse 11/1/2, Vienna, 1190 Austria

**Keywords:** Lipid bilayer, Methyl-β-cyclodextrin, Cholesterol extraction, Atomic force microscopy, Quartz crystal microbalance with dissipation

## Abstract

This study investigated the biophysical impacts of methyl-β-cyclodextrin (MβCD) on supported lipid bilayers composed of DPPC/POPE (1:1) and DPPC/POPE/Chol (1:1:2). Using Quartz Crystal Microbalance with Dissipation (QCM-D), Atomic Force Microscopy (AFM) imaging and force–distance (F–D) curve analysis, we monitored vesicle fusion, assessed bilayer symmetry, and evaluated mechanical stability before and after MβCD treatment. The DPPC/POPE bilayer initially showed a distinct “kink” in its F–D curve and a single breakthrough force, indicating a relatively asymmetric and heterogenous structure. Upon MβCD treatment, the bilayer exhibited symmetry, as evidenced by breakthrough force profiles. Similarly, the cholesterol-containing DPPC/POPE/Chol bilayers displayed two breakthrough points, signifying distinct lipid domains, higher rigidity, and heterogeneity. After one hour of MβCD treatment, these bilayers became more homogeneous and showed a single breakthrough peak—evidence of successful cholesterol or phospholipid extraction. Breakthrough force histograms reinforced these findings by revealing substantial shifts in force distributions. Complementary dynamic light scattering (DLS) and zeta potential measurements confirmed vesicle size and surface charge, while QCM detected notable mass loss in cholesterol-rich bilayers at elevated temperatures. Collectively, these findings underscore the role of cholesterol in bilayer architecture and validate MβCD as an effective modulator of bilayer composition and symmetry.

## Introduction

Understanding how membranes respond to various physical and chemical stimuli is essential for elucidating the mechanisms behind some biological processes such as membrane trafficking, signal transduction, cell fusion, and antimicrobial defense (Klebe and Bentley 1987; Mishev et al. 2013; Bechinger 2015; Thompson and Chan 2016). The cell membrane’s complex behavior is influenced by its molecular composition and dynamic organization (van Meer et al. 2008; Aguilera-Romero et al. 2023). To study these behaviors in a simplified and controllable context, supported lipid bilayers (SLBs) are widely used as model membrane systems. These artificial bilayers replicate many fundamental properties of biological membranes, including lateral fluidity, phase separation, and lipid packing, while offering compatibility with high-resolution biophysical techniques (Glazier and Salaita 2017; Mangiarotti et al. 2025). SLBs enable systematic investigation of how membrane structure and function are influenced by specific components such as phospholipids, cholesterol, and interacting molecules.

Perturbation of SLBs can be triggered by various agents, including peptides, proteins, detergents, and small molecules (Hammond et al. 2021; Ammendolia et al. 2021). One widely studied form of perturbation involves the disruption of membrane integrity. This is a process often initiated by interactions that destabilize lipid packing or extract membrane components. Among these, changes in cholesterol content are particularly impactful, as cholesterol plays a critical role in modulating bilayer stiffness, phase behavior, domain formation, and protein-lipid interactions (Tharad et al. 2018; Goh and Tero 2021; Doole et al. 2022). Depletion of cholesterol can lead to compromised membrane order and increased susceptibility to deformation or disintegration (Hao et al. 2004; Wheeler and Tyler 2011). One commonly used agent to remove cholesterol is methyl-β-cyclodextrin (MβCD), which acts by selectively extracting cholesterol from membranes. MβCD has been extensively applied in cellular studies to investigate cholesterol’s functional roles. However, its use in simplified model systems particularly SLBs has been comparatively limited. Such systems offer a valuable opportunity to gain mechanistic insight into cholesterol-driven membrane organization and stability (Gunasekara et al. 2010; Abe and Kobayashi 2021; Reagle et al. 2024).

Prior works in model membranes, especially vesicles, have demonstrated that the interaction of MβCD with lipids depends strongly on the phospholipid headgroup, acyl chain composition, and MβCD concentration. The efficiency of lipid extraction by MβCD increases with shorter and more fluid acyl chains (Anderson et al. 2004; Bozelli et al. 2017; Markones et al. 2018). In particular, the work by Markones et al. (2018) on stoichiometry and the effect of symmetric versus asymmetric lipid compositions provide a valuable framework for understanding how phospholipid removal may differentially destabilize bilayers with mixed phospholipid content (Markones et al. 2018).

Despite these advances, most prior investigations of MβCD’s effects have focused on vesicle-based systems, while studies examining its impact on supported lipid bilayers (SLBs) using atomic force microscopy (AFM) remain comparatively limited. In this work, we investigate the effect of MβCD on SLBs with and without cholesterol. Atomic force microscopy (AFM) is widely used for direct visualization of membrane topography as well as to study membrane mechanics at nanometer resolution. It enables the detection of physical changes such as pore formation, lipid domain disintegration, thinning of bilayers, or the appearance of nanoscale defects resulting from cholesterol extraction or other perturbative events (Green et al. 2004; Juhaniewicz and Sek 2015; Aryal and Pan 2024). Beyond static imaging, AFM can also provide force distance measurements that provide bilayer stiffness and mechanical resistance—important parameters that change in response to physical stress or biochemical perturbation (Garcia-Manyes and Sanz 2010; Griffo et al. 2024). These capabilities make AFM a valuable tool for characterizing the structural and mechanical consequences of MβCD-mediated cholesterol depletion in SLBs.

Complementing AFM, quartz crystal microbalance with dissipation monitoring (QCM-D) provides quantitative insight into the mass and viscoelastic properties of lipid bilayers in real time. This technique measures differences in oscillation frequency and energy dissipation of a quartz crystal as a function of molecular adsorption or structural changes on its surface. In the context of membrane disruption, QCM-D can detect events such as vesicle adsorption, partial bilayer formation, loss of lipid material, or softening of the membrane structure. It has been successfully applied to study how toxins, or protein-lipid interactions destabilize bilayers (Nielsen and Otzen 2013; Tharad et al. 2015, 2017). By combining AFM’s spatial resolution with QCM-D’s sensitivity to dynamic mass and viscoelastic changes, researchers can build a detailed understanding of how membranes undergo structural failure or rearrangement under different perturbative conditions.

The aim of this study is to investigate the structural and mechanical responses of model lipid bilayers to phospholipid/cholesterol depletion induced by MβCD, using a multi-method approach. To achieve this, we employ three complementary biophysical techniques—AFM, QCM-D, and dynamic light scattering (DLS)—to monitor changes at different physical scales. AFM is used both for high-resolution imaging of supported lipid bilayers and for collecting force–distance (F–D) curves, which provide information on the nanomechanical properties. These measurements help visualize surface defects, thinning, or domain reorganization and quantify how resistant the bilayer is to deformation. QCM-D is applied to detect changes in the viscoelastic properties and mass of the bilayer during disruption, which can reveal whether lipid material is being removed from the surface or whether the bilayer becomes softer and less rigid. In parallel, DLS is used to monitor liposome size and its change in suspension before bilayer formation, providing insight into how disruption processes affect the size distribution of lipid assemblies. Together, these methods offer a comprehensive picture of membrane disruption, integrating topographical, mechanical, and dynamic measurements to better understand how lipid bilayers respond to perturbation.

## Materials and methods

### Materials

All lipids were purchased from Avanti Polar-Lipids (Alabaster, US) including 1,2-dipalmitoyl-phosphatidylcholine (DPPC), 1-palmitoyl-2-oleoyl-sn-glycero-3-phosphoethanolamine (POPE), and cholesterol. They were dissolved in methanol: chloroform (1:4). Phosphate-Buffered Saline (PBS) 1X was prepared by dissolving a PBS tablet (Carl ROTH) in 500 mL mili-Q water (resistivity 18.2 MΩ·cm). Methyl-β-cyclodextrin (MβCD) was obtained from Sigma Aldrich (Darmstadt, Germany).

### Liposome Preparation

Two different lipid systems were prepared: DPPC/POPE (1:1 molar ratio) and DPPC/POPE/Chol (1:1:2 molar ratio). All lipids were dissolved in a chloroform: methanol mixture (4:1). The organic solvent was evaporated under a nitrogen stream until it had completely evaporated. To prepare a 1 mg/mL liposome solution, 1 mL of PBS solution was poured into a tube and vortexed for 2–3 min. Sonication was then used (5–10 min) at 50 °C to homogenise the solution. The liposomes were then extruded 25 times through a 50 nm polycarbonate membrane at 50 °C using a mini extruder (Avanti, Alabaster, USA). The liposome suspension was flushed with nitrogen to prevent oxidation and stored at 4 °C. While the initial molar ratio of cholesterol was 50%, we note that the actual cholesterol content in the bilayer may be slightly lower due to the known limitations of solvent-based vesicle preparation. Extrusion likely homogenizes the vesicles, minimizing the impact of any cholesterol precipitates, although our measurements are not sensitive to small amounts of excess cholesterol crystals (Huang et al. 1999; Day et al. 2002; Vist and Davis 2002; Mannock et al. 2003).

### Liposome characterization

The size and zeta potential of the liposomes were measured using Zetasizer Nano ZS (Malvern Instrument, Worchestershire, UK). The untreated 0.1 mg/mL liposome suspension was prepared from the stock for both measurements. A 100 µL of MβCD 2.5 mM in PBS was added into 1 mL untreated liposome 0.1 mg/mL and left for roughly 10 min to figure out the evolution of liposome size. The size of untreated and MβCD -treated liposomes was then determined by replicating the experiments three times.

### Quartz crystal microbalance with dissipation (QCM-D) measurement

QCM-D experiments were performed using Q-Sense E4 Instrument (Q-Sense AB, Sweden) to observe lipid bilayer formation and disruption, adapted from (Tharad et al. 2015). Prior to measurement, silicon chips (QSX 303, 4.95 MHz, Q-Sense AB) were sonicated in 2% w/v SDS solution (15 min), water (15 min), and rinsed with ethanol. Afterwards, the chips were dried with nitrogen stream and cleaned with UV/ozone (Bioforce Nanoscience, USA) for 15 min. The QCM chips were then mounted in QCM chambers and temperature of the experiment was set at 25 °C, 30 °C, and 45 °C. The chamber was then filled with PBS 1X (pH 7.4) at a flow rate of 50 µL/min using a peristaltic pump (SM935C, Ismatec, Zurich, Switzerland); a stable baseline was achieved after approximately 15 min of equilibration. Prior to measurement, the frequency overtone was determined and recorded at the corresponding temperature. To investigate the lipid bilayer by vesicle fusion, 0.1 mg/mL of liposomes were injected into the chamber at a rate of 50 µL/min via a peristaltic pump until a stable signal was obtained, with the temperature being monitored throughout. Residual liposomes were removed by rinsing the system using PBS through the chamber at a similar flow rate for roughly 30 min. Each injection was recorded.

At the same temperature and flow rate, a 2.5 mM MβCD solution was subsequently introduced into the chamber for 5 min to monitor lipid detachment. After injection, one-hour incubation period was applied to enable MβCD-bilayer interaction. All experiments were conducted and recorded in various frequency overtone (Δf) and dissipation (ΔD) (*n* overtone = 1, 3, 5, …11). Afterwards, to confirm complete lipid depletion, PBS was reintroduced into the chamber until a stable baseline was re-established.

The Sauerbrey equation (*Δm=-(C/n)·Δf*) relates the adsorbed mass (*Δm*) on a crystal surface proportionally as a function of the frequency change (*Δf*). In the equation (*Δm*) represents the mass surface density (ng cm^− 2^), *(C)* is a proportionality constant that depends only on the intrinsic properties of the sensor (17.7 ng Hz^− 1^ cm^− 2^, for this AT-cut quartz sensor), (*Δf*) is the frequency shift, and *(n)* is the overtone number. This model assumes that the film on the surface is rigid, which is validated by low energy dissipation values (*D*), indicating minimal viscoelastic effects. When *D* is high, it suggests a soft, dissipative film, and the Sauerbrey model no longer applies, requiring more complex analysis. In this case, the system’s (*ΔD/Δf*) ratio remains below 0.2 × 10⁻⁶, indicating that the Sauerbrey model is valid for the measurements (Glasmästar et al. 2002). Furthermore, the *ΔD/Δf* plot provides insights into the time-resolved viscoelastic properties of the film as its mass changes.

It should be noted that all solutions used were placed on a hotplate to adjust the temperature to the selected values during measurement, while the QCM-D chamber was set up accordingly. At least three replications were conducted to ensure reproducibility. Mass adsorption and layer thickness were calculated automatically using Q-Tools software.

### AFM observation

A SPM Multimode (Veeco, Bruker, US) was used to observe liposome appearance, the formation of bilayers and their defects, and to perform force-distance (F-D) measurements. For imaging in tapping mode, we used SNL-10 tip cantilever (Bruker, California, US) with a 2 nm radius and spring constant of 0.24 N/m. The scan rate was between 1 and 2 Hz, scan size of 5 μm, and the resolution was 512 × 512 pixel. For F-D measurements, at least 55 points of measurement were collected from three different spots.

Muscovite mica was used for lipid vesicle deposition. Before imaging the liposome morphology, the mica was cleaned using Milli-Q water and treated with UV/ozone for a duration of 15 min, while the liposome solution was cooled to 5–10 °C (to prevent vesicle fusion during deposition). A 25 µL of a 0.1 mg/mL liposome solution was dropped onto the mica surface and immediately dried with a nitrogen stream. The deposited liposomes were then characterised using AFM in tapping mode. This was also done for MβCD -treated liposomes.

To capture bilayer formation, 150 µL of liposome 0.1 mg/mL was injected into the AFM chamber that previously was heated up to 55 °C. Beforehand, the AFM sample holder was rinsed using PBS pH 7.4. Bilayer formation was captured in 15, 30, and 45 min at scan rate of 1–2 Hz. After one hour, the chamber was flushed using PBS to remove residual liposome and observe the F-D curve. Subsequently, 150 µL MβCD 2.5 mM was injected into chamber and recorded at the same interval time to figure out the lipid depletion. We flushed the chamber with PBS to ensure observation of the bilayer before and after treatment only and continued with F-D measurement. The AFM images were analyzed using Nanoscope Analysis 3.0 (Bruker, CA) and while F-D curves were analyzed using Python 3.13.

## Results and discussion

### Liposome characterization

**Fig. 1:**
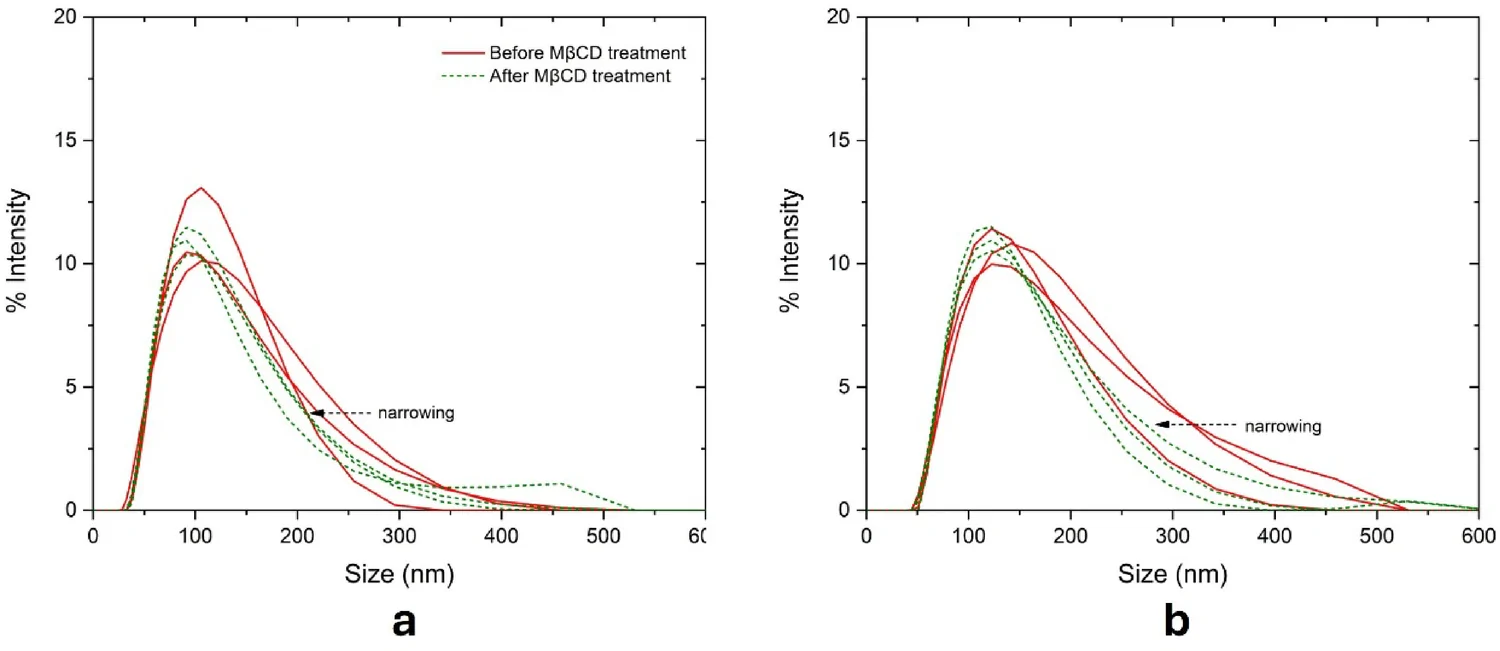
Dynamic light scattering (DLS) profiles of bilayer lipid vesicles before and after MβCD treatment. (**a**) DPPC/POPE (1:1); (**b**) DPPC/POPE/Chol (1:1:2). The red solid lines represent the size distribution before MβCD treatment, and the green dashed lines represent the distribution after 10 min of MβCD administration. The shift towards smaller particle sizes (indicated by the arrows) suggests narrowing distribution, which is likely due to lipid reorganization or extraction. This is particularly evident in the cholesterol-containing formulation (b). The experiments were performed at 25 °C for all systems

Evaluating the disruption liposome becomes an interesting topic since it can be done in multiscale characterization. One of the easiest ways to scrutinize this phenomenon is to use DLS. We evaluate how liposomes behave in the presence of MβCD. The size distribution of liposomes is shown in Fig. 1.

The vesicles prior to treatment (red curves) displayed broad size distribution peaking around ~ 100–110 nm. Although the extrusion process using 50 nm membrane was intended to homogenize the vesicle population, DLS results revealed some size heterogeneity. This suggests the coexistence of unilamellar and multilamellar vesicles. In addition, the relatively low surface charge of the vesicles may promote aggregation. Increasing the ionic strength of the solvent reduces interbilayer repulsion, thereby decreasing the formation of unilamellar vesicles after extrusion (Scott et al. 2019).

The reduction in vesicle size was more pronounced in the cholesterol-containing formulation (Fig. 1b) after MβCD administration. This effect results in reduced particle size, likely due to decreased membrane fluidity and vesicle collapse. In contrast, the cholesterol-free DPPC/POPE vesicles (Fig. 1a) also experienced size reduction, although to a lesser extent. Unlike the results reported by Bozelli et al. (2017), where high MβCD concentrations (60 mM) and low lipid/MβCD molar ratios (< 0.01) led to complete vesicle solubilization, we employed our experiments a lower MβCD concentration (2.5 mM), corresponding to a lipid/MβCD ratio of approximately 0.55. At this ratio, MβCD is present in limiting amounts, and full disruption of the bilayer is not expected. Consequently, DLS measurements showed no statistically significant changes in the average vesicle size (p-value = 0.23 for DPPC/POPE and p-value = 0.76 for DPPC/POPE/Chol). However, the size distribution narrowed slightly after MβCD treatment (Fig. 1; Table 1), likely reflecting partial extraction of accessible surface lipids or cholesterol. The subtle difference between DPPC/POPE and DPPC/POPE/Chol systems further supports the preferential removal of cholesterol under sub-solubilizing MβCD conditions. Overall, these minor changes indicate slight vesicle surface remodeling, while the average vesicle size remains statistically unchanged.

**Fig. 2:**
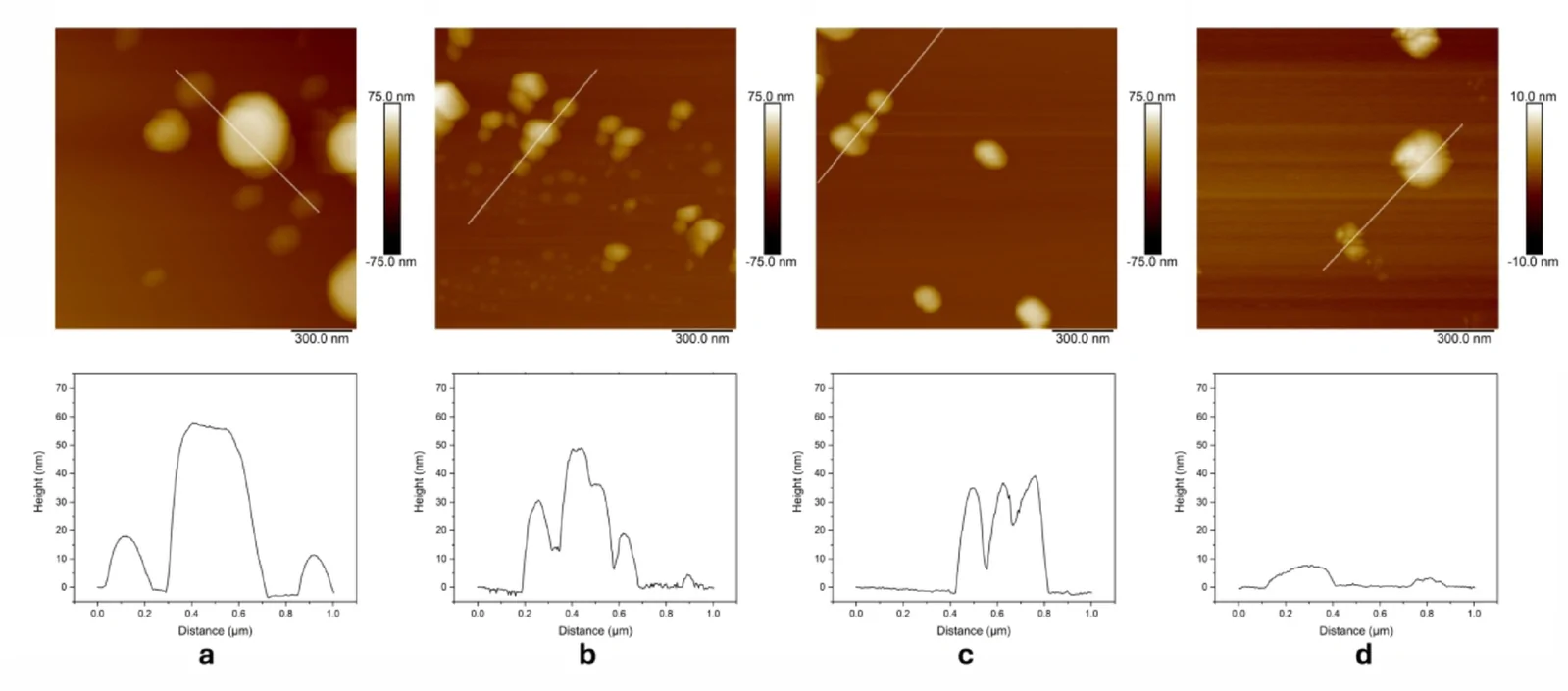
Atomic Force Miscroscopy (AFM) topographical images and corresponding height for lipid vesicles before and after MβCD administration. (**a**) DPPC/POPE (1:1) before MβCD treatment; (**b**) DPPC/POPE (1:1) after a 10-minute MβCD treatment; (**c**) DPPC/POPE/Chol (1:1:2) before treatment; (**d**) DPPC/POPE/Chol (1:1:2) after a 10-minute MβCD treatment. All scale bars represent 300 nm. Color height scales range from − 75 nm to 75 nm for (a–c), and from − 10 nm to 10 nm for (d)

AFM images revealed gradual morphological changes in the supported lipid structures following MβCD treatment. Prior to MβCD treatment, the DPPC/POPE (1:1) liposomes (Fig. 2a) appear as spherical protrusions ranging in height from ~ 20 nm to ~ 60 nm and in size from ~ 170 nm to ~ 400 nm. Incorporating cholesterol into the liposome formulation (DPPC/POPE/Chol, 1:1:2) results in fewer, more uniform spherical particles (Fig. 2c). If we scrutinize DLS measurements, these cholesterol-containing liposomes exhibited slightly larger hydrodynamic diameters than the cholesterol-free system, consistent with the stabilizing effect of cholesterol. In the AFM images, the DPPC/POPE/Chol vesicles generally appear brighter in the color scale, indicating greater relative thickness compared to DPPC/POPE vesicles, which result in an increased rigidity and structural stability, despite some aggregation observed in the DPPC/POPE sample.

Figure 2b shows that MβCD treatment results in a noticeable change in the morphology of DPPC/POPE (1:1) liposomes. The number of particles increases, but their size decreases. The height profile shows peaks mostly between ~ 20 and 50 nm, indicating partial liposome disruption or shrinkage. This may be due to MβCD interacting with the lipid components, which could lead to the partial solubilization or destabilization of the vesicle membranes. Additionally, the vesicle structures became more fragmented.

Furthermore, cholesterol-rich liposomes (Fig. 2d) show a dramatic reduction in size and topographical features. The particles appear flatter and shorter, with a profile showing features below ~ 10 nm. This indicates that MβCD effectively extracts cholesterol (and phospholipid) from the liposomes, resulting in membrane collapse or fragmentation.

Such morphological changes are consistent with the DLS results presented earlier, which showed a shift towards smaller hydrodynamic diameters following MβCD treatment. Although MβCD is known to preferentially remove cholesterol from cellular matrices or model systems (Sanchez et al. 2011; Biswas et al. 2019; Abe and Kobayashi 2021), it can also remove phospholipids or fatty acids from vesicle matrices (Leventis & Silvius, 2001; Monnaert et al. 2004; Brunaldi et al.^ 2010^; Reagle et al. 2024). Consequently, structural changes can still be observed in the DPPC/POPE (1:1) system during MβCD treatment.

### QCM study of lipid bilayer formation and depletion

QCM is a versatile tool for lipid bilayer formation via vesicle fusion. The experiment was conducted at three different temperatures, i.e., 25 °C, 30 °C, and 45 °C as the properties of selected lipids, i.e. DPPC has a transition temperature at 41 °C while POPE has a transition temperature at 25 °C. The QCM study was started by the injection of PBS to find the baseline, followed by liposome injection (and bilayer formation). PBS was then injected to release the residual lipids. To study the impact of MβCD, the lipids were exposed to it for one hour and then flushed with PBS to ensure lipid depletion.

**Fig. 3:**
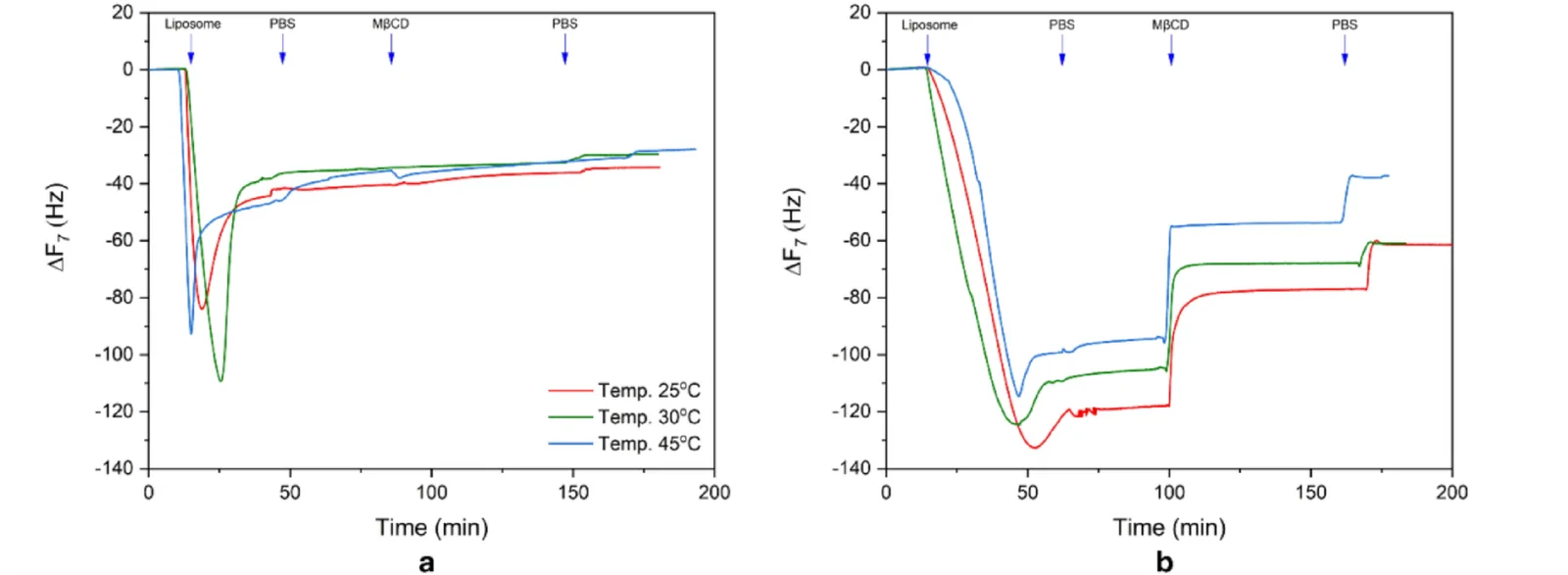
Quartz crystal microbalance (QCM) analysis of supported lipid bilayer at different temperatures. (**a**) DPPC/POPE (1:1) and (**b**) DPPC/POPE/Cholesterol (1:1:2). The data represented are based on the seventh overtone. The red, green and blue lines represent experiments conducted at 25 °C, 30 °C and 45 °C, respectively. The arrows indicate the sequential addition of liposomes, PBS, MβCD and the final PBS wash

The QCM-D data presented in Fig. 3 illustrate the influence of temperature on the fusion and bilayer formation of vesicles composed of (a) DPPC/POPE (1:1) and (b) DPPC/POPE/Chol (1:1:2), respectively. In Fig. 3a, all selected temperatures facilitate vesicle adsorption and subsequent fusion, as evidenced by the initial sharp decrease in frequency (ΔF₇). The rupture releases water mass and lipids generating the increase in frequency. A final PBS rinsing ensures removing residual lipids or weakly attached particles from bilayer systems (Swana et al. 2022). Subsequent MβCD treatment gradually releases lipids from the system, as indicated by a slight increase in frequency to approximately − 36 Hz, − 33 Hz, and − 37 Hz at 25 °C, 30 °C, and 45 °C, respectively. The final frequency shifts observed after PBS rinsing suggest the removal of phospholipids from the bilayer systems, although the increase is minimal—less than 5 Hz compared to the preceding measurements.

In contrast, Fig. 3b shows a markedly different behavior with high cholesterol content in the lipid composition. At all selected temperatures, vesicle fusion is slower and results in deeper frequency shifts. It seems that vesicles did not experience two similar steps as DPPC/POPE (1:1), including vesicle absorption and rupturing. Instead, the frequency dropped continued with stabilization only until − 121 Hz (25 °C), -106 Hz (30 °C), and − 95 Hz (45 °C) even after first PBS rinse. The formation of the bilayer depends on both the substrate charge and the surface charge of the vesicles. Since both the SiO₂ QCM chip and the bilayer vesicles carry negative charges, adsorption onto the substrate is slightly hindered. This is further supported by the observation that, in some systems, the lipid composition does not reach the transition temperature, limiting complete adsorption. After adsorption, the frequency shift increases (indicating mass loss) and the dissipation decreases to a small plateau, consistent with vesicle fusion and rupture, which releases entrapped water and forms a thin, rigid bilayer, as reported previously (Keller and Kasemo 1998; Bar et al. 2022). The rupture of vesicle happens spontaneously after adsorption, even though it is only partial.

**Fig. 4:**
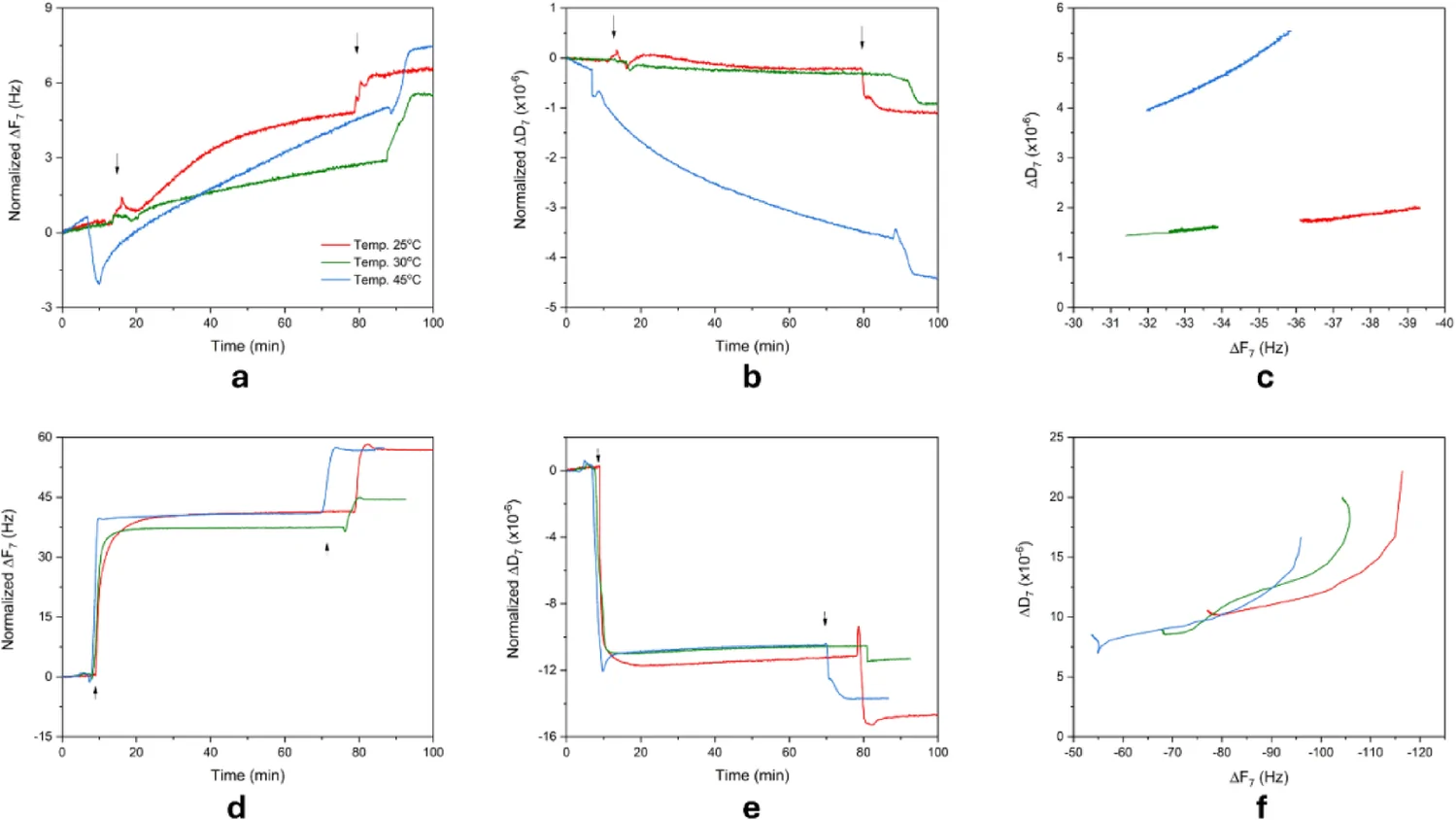
MβCD-lipid bilayer interaction at different temperatures. (Top) DPPC/POPE (1:1) and (bottom) DPPC/POPE/Chol (1:1:2). Panels (**a**–**c**) show the response of DPPC/POPE bilayers: (**a**) normalized frequency (ΔF₇) versus time, (**b**) normalized dissipation (ΔD₇) versus time, (**c**) ΔD₇ versus ΔF₇ plots. Panels (**d**–**f**) correspond to DPPC/POPE/Chol bilayers: (**d**) normalized frequency vs. time; (**e**) normalized dissipation vs. time; and (f) ΔD₇ vs. ΔF₇ plots. All experiments were conducted at the respective temperatures of 25 °C, 30 °C and 45 °C. Arrows indicate the injection of MβCD (left arrow) and subsequent PBS rinsing (right arrow) after bilayer stabilization. The data illustrate the temperature- and cholesterol-dependent effects of MβCD on bilayer mass and viscoelastic properties

Figure 4 shows the QCM analysis after MβCD injection, illustrating the changes in frequency and dissipation. This figure demonstrates how the viscoelastic properties of the bilayer systems are altered following MβCD treatment. To note, the images are normalized frequency and dissipation, not factual raw data. Therefore, the frequency changes have positive values while dissipations have negative values. In the cholesterol-free DPPC/POPE system (Fig. 4a-c), a small but consistent frequency increase is observed upon MβCD injection at all temperatures, suggesting gradual lipid removal. The shift is modest compared to cholesterol-containing bilayers (Fig. 4d-f). This observation indicates that MβCD interacts weakly with phospholipids alone. Notably, the frequency shift is largest at 45 °C, implying enhanced lipid mobility and extraction at higher temperatures. This is supported by the dissipation data (panel b). The curve shows a noticeable decrease, especially at elevated temperature, suggesting bilayer thinning or loosening due to partial lipid extraction.

The ΔD₇ versus ΔF₇ plots (panel c) for the DPPC/POPE system exhibit subtle, near-linear trends across all examined temperatures, indicating a structurally stable bilayer with only minimal mass loss and viscoelastic alteration. These observations support the notion that MβCD is capable of extracting phospholipids from the bilayer; however, its interaction remains limited when cholesterol is absent. In case of bilayer containing DPPC which has saturated acyl chain, it needs relatively high concentration of MβCD to detach from the system (Huang and London 2013).

In contrast, the DPPC/POPE/Chol bilayers (Fig. 4d–f) display a markedly different response. After MβCD administration, all temperatures show substantial frequency increases (~ 40–50 Hz), indicating of substantial mass removal. Interestingly, while the largest ΔF₇ shift occurs at 45 °C, the 25 °C and 30 °C bilayers also exhibit strong responses. This demonstrates that cholesterol extraction occurs efficiently across this temperature range. Figure 4e highlights the corresponding dissipation changes. Upon cholesterol removal, the bilayers become more rigid, shown by the reduction in ΔD₇ following MβCD injection. After PBS rinsing, a slight dissipation increase is seen, especially at higher temperatures, possibly due to residual phospholipid loss or destabilization of the bilayer architecture.

The ΔD₇ vs. ΔF₇ plots for cholesterol-rich bilayers (panel f) reveal nonlinear trajectories, in sharp contrast to the more linear behavior observed in the cholesterol-free system. The data suggest that MβCD treatment induces significant membrane restructuring, particularly in cholesterol-rich systems. Notably, there is a proportional relationship between decreasing frequency (indicative of mass uptake) and increasing dissipation. Or in other words, when mass is reduced—i.e., when frequency increases—dissipation tends to decrease, indicating that the bilayer becomes more rigid and less hydrated after lipids removal. This dynamic process is consistent with the mechanism described by Cho et al. (2009), where vesicle transformation involves several stages: initial intact vesicle interaction, MβCD contact with the bilayer surface, vesicle rupture, and eventual formation of a rigid planar bilayer. The frequency and dissipation shifts observed here are attributed to these stages, in particular the disruption and reorganization of vesicle layers during MβCD treatment (Cho et al. 2009). Additionally, vesicle destabilization leads to a significant uptake of hydrodynamically coupled solvent mass. This increase in film hydration is further supported by the corresponding rise in energy dissipation. Similar phenomena have been reported in other systems, such as α-helical peptide interacting with vesicle layer (Chahw et al. 2008; Cho et al. 2009; Zan et al. 2014; Zan and Cho 2014).

At the right end of the curve, the dissipation graph appears to increase while the frequency remains stable. However, upon closer inspection, the graph is not entirely constant but shows slight variations. This graph corresponds to the measurement taken after the first rinsing with PBS, right before the lipid bilayer was introduced with MBCD. Therefore, in terms of scale, the frequency and dissipation values at the right end of the curve — for the blue, green, and red curves in panel f — are proportional.

**Table 1:** The characteristics of liposomes and lipid bilayers (*n* > 3)

Liposome	Size based on DLS (nm)		*p* value based on paired t-test*	Zeta potential (mV)	% Mass reduction based on QCM		
	Before	After 10 min MβCD			25 °C	30 °C	45 °C
DPPC/POPE (1:1)	119.6 ± 53.8	116.4 ± 55.6	0.23	-7.4 ± 0.5	14.9 ± 1.0	7.2 ± 2.4	15.2 ± 3.5
DPPC/POPE/Chol (1:1:2)	157.5 ± 56.0	142.0 ± 67.0	0.76	-4.9 ± 0.3	50.7 ± 2.6	46.1 ± 3.3	60.1 ± 0.3

* Confidence Interval is 95%

Using DLS, zeta potential and QCM-D, we observed the effect of MβCD treatment on DPPC/POPE (1:1) and DPPC/POPE/Chol (1:1:2) liposomes (Table 1). Only minor changes in vesicle size were detected by DLS after 10 min of MβCD exposure. Statistical analysis confirmed that these differences were not significant (p value = 0.23 for DPPC/POPE and p value = 0.76 for DPPC/POPE/Chol), indicating that MβCD treatment does not substantially alter vesicle size or integrity in bulk suspension. However, zeta potential analysis revealed that DPPC/POPE vesicles were more negatively charged than those containing cholesterol. The reason behind this is cholesterol intercalates between phospholipid molecules, producing a tighter and more ordered membrane structure, which limits the accessibility of charged lipid headgroups to surrounding ions (Magarkar et al. 2014; Banerjee et al. 2023). This reduced ion binding at the membrane surface leads to a smaller effective surface charge. Such condition results in a decrease in the measured zeta potential. In anionic membranes, cholesterol similarly reduces the magnitude of the negative zeta potential due to its influence on membrane electrostatics.

The QCM data provides compelling evidence of MβCD-mediated lipid extraction, particularly at high temperatures. DPPC/POPE/Chol (1:1:2) shows a significantly greater mass reduction than DPPC/POPE (1:1) at all temperatures, exceeding 60% at 45 °C. This temperature-dependent mass loss suggests that MβCD selectively extracts cholesterol and that higher temperatures increase lipid mobility and destabilization. In contrast, DPPC/POPE (1:1) bilayers demonstrate relatively low mass reduction, indicating that MβCD interacts less efficiently with cholesterol-free membranes. While the reduction percentages are different, these findings are consistent with each other.

### AFM study of lipid bilayer

**Fig. 5:**
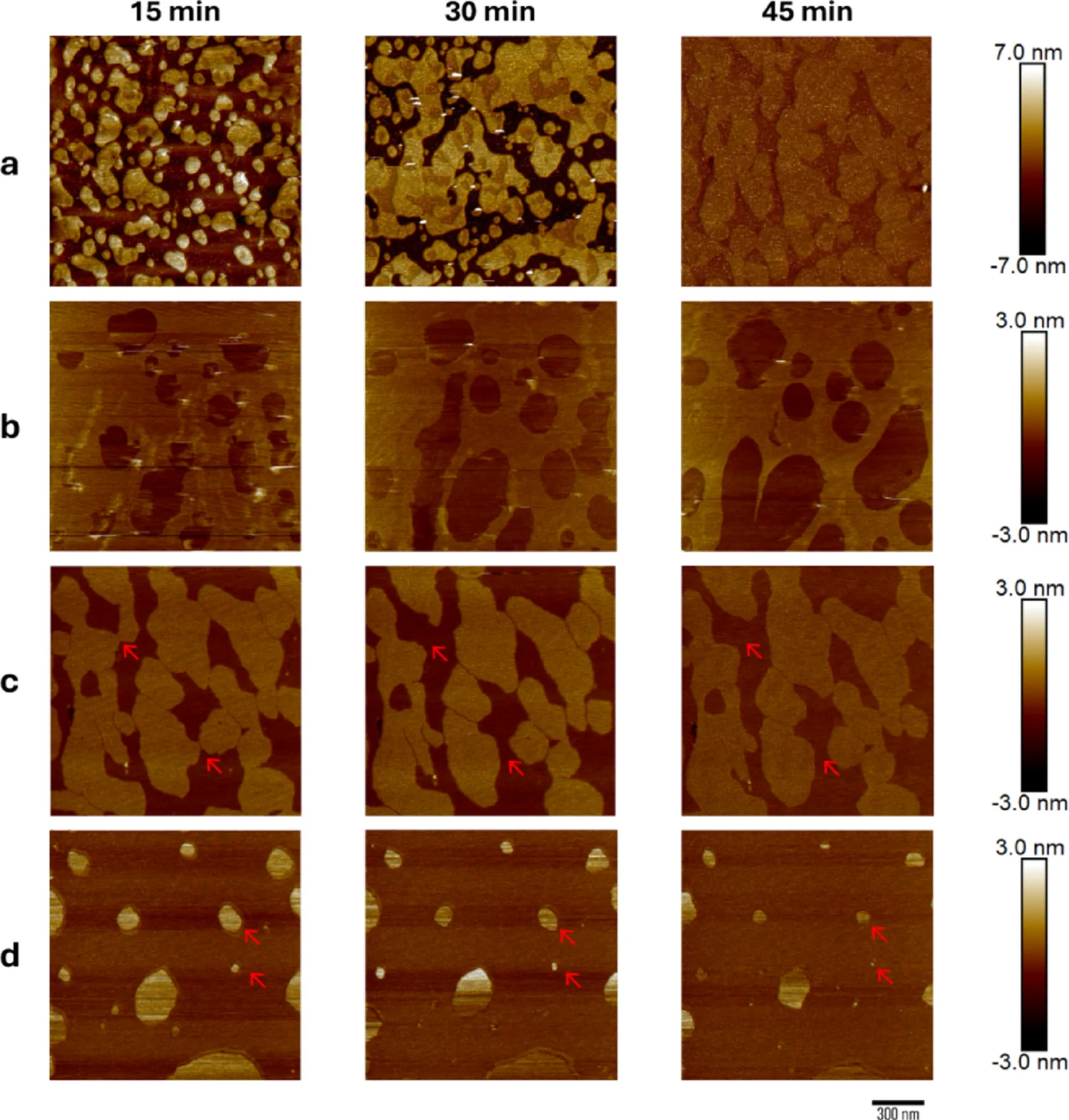
AFM topographic images showing vesicle fusion and MβCD-induced disruption in supported lipid bilayers. (**a**) The time course of DPPC/POPE (1:1) bilayer formation over 30 min, showing progressive bilayer formation alongside persistent domain boundaries and fusion defects. (**b**) The formation of a DPPC/POPE/Chol (1:1:2) bilayer over 15–45 min, showing enhanced domain coalescence and smoother formation of the bilayer due to the presence of cholesterol. (**c**) Disruption of a preformed DPPC/POPE bilayer following MβCD treatment, showing visible defects and sites of lipid extraction (red arrows). (**d**) MβCD treatment of the DPPC/POPE/Chol (1:1:2) bilayer, which leads to the pronounced disintegration of domains and the removal of cholesterol (red arrows). The height scales for each row are indicated on the right

Figure 5 displays atomic force microscopy (AFM) images capturing the formation and disruption of model membrane bilayers. The panels are arranged to represent four distinct experimental conditions; each tracked over time and following specific treatments. The first two columns (a and b) illustrate the time-resolved formation of bilayers for the DPPC/POPE (1:1) and DPPC/POPE/Chol (1:1:2) systems, respectively. Columns c and d display the corresponding bilayer structures following treatment with MβCD. Each row represents a sequential time point or stage in the treatment process. The adjacent color bars indicate nanoscale height variations, reflecting surface topography across the bilayer.

**Table 2:** Surface coverage of bilayer formation and after MβCD-treatment

Time	Surface Coverage (%)			
	Untreated		MβCD-treated	
	DPPC/POPE (1:1)	DPPC/POPE/Chol (1:1:2)	DPPC/POPE (1:1)	DPPC/POPE/Chol (1:1:2)*
15 min	44.0 ± 2.8	70.1 ± 9.3	65.1 ± 0.1	15.5 ± 3.6
30 min	69.3 ± 5.2	68.1 ± 6.5	58.1 ± 2.9	10.2 ± 3.3
45 min	73.8 ± 0.2	59.4 ± 7.6	57.5 ± 3.5	8.4 ± 3.7

*Calculated from protrusion only

Figure 5a shows that the fusion of DPPC/POPE (1:1) vesicles results in a progression from a rough, granular surface after 15 min to a more continuous bilayer after 45 min. This pattern of domain formation is consistent with the known immiscibility between DPPC and POPE. In DPPC/POPE or DPPC/POPE/Chol systems, the domains are composed of DPPC surrounded by POPE- or POPE/Chol-rich regions (Garvey et al. 2023). Figure 5b, which includes cholesterol, shows that domain formation is more rapid and uniform after 30 min and that the bilayer is more stable and ordered after 45 min. Cholesterol appears to promote domain coalescence, resulting in a smoother surface with no visible domain boundaries.

Figure 5c presents the DPPC/POPE (1:1) bilayer after MβCD treatment. The red arrows indicate regions from which material has been extracted, resulting in the formation of pits or domain loss. This suggests that MβCD disrupts membrane integrity by either solubilizing lipids or disturbing domain interactions. Similarly, panel (d) shows the DPPC/POPE/Chol (1:1:2) bilayer after MβCD treatment. Although cholesterol generally stabilizes bilayers, MβCD’s affinity for cholesterol causes pronounced extraction and thinning of lipid domains (again marked by arrows).

Overall, this figure effectively illustrates the dynamic processes involved in bilayer formation, as well as the disruptive potential of MβCD on lipid membranes. It shows how cholesterol modulates bilayer morphology and enhances fusion efficiency to form stable domains but also becomes vulnerable when targeted by cyclodextrin.

Quantitative analysis of surface coverage, presented in Table 2, corroborates these topographical observations. In the untreated DPPC/POPE (1:1) system, surface coverage increases from 44.0 ± 2.8% after 15 min to 73.8 ± 0.2% after 45 min, which indicates that the vesicles spread effectively and form a bilayer over time. In the untreated DPPC/POPE/Chol (1:1:2) system, initial coverage is high at 70.1 ± 9.3% after 15 min and decreases slightly to 59.4 ± 7.6% after 45 min. This trend may reflect minor domain rearrangement or detachment, but it still indicates that cholesterol facilitates more rapid and efficient bilayer formation.

In contrast, MβCD-treated systems show markedly reduced bilayer formation. The DPPC/POPE (1:1) bilayer treated with MβCD exhibits 65.1 ± 0.1% coverage at 15 min, with only a modest change to 57.5 ± 3.5% at 45 min, suggesting that MβCD impairs membrane integrity and limits bilayer expansion. The most pronounced effect is observed in the MβCD-treated DPPC/POPE/Chol (1:1:2) system, where surface coverage by protrusion is only 15.5 ± 3.6% at 15 min and declines further to 8.4 ± 3.7% by 45 min. This dramatic loss supports the visual observation of cholesterol extraction and structural disruption seen in Fig. 5d.

### Nanomechanical properties

**Fig. 6:**
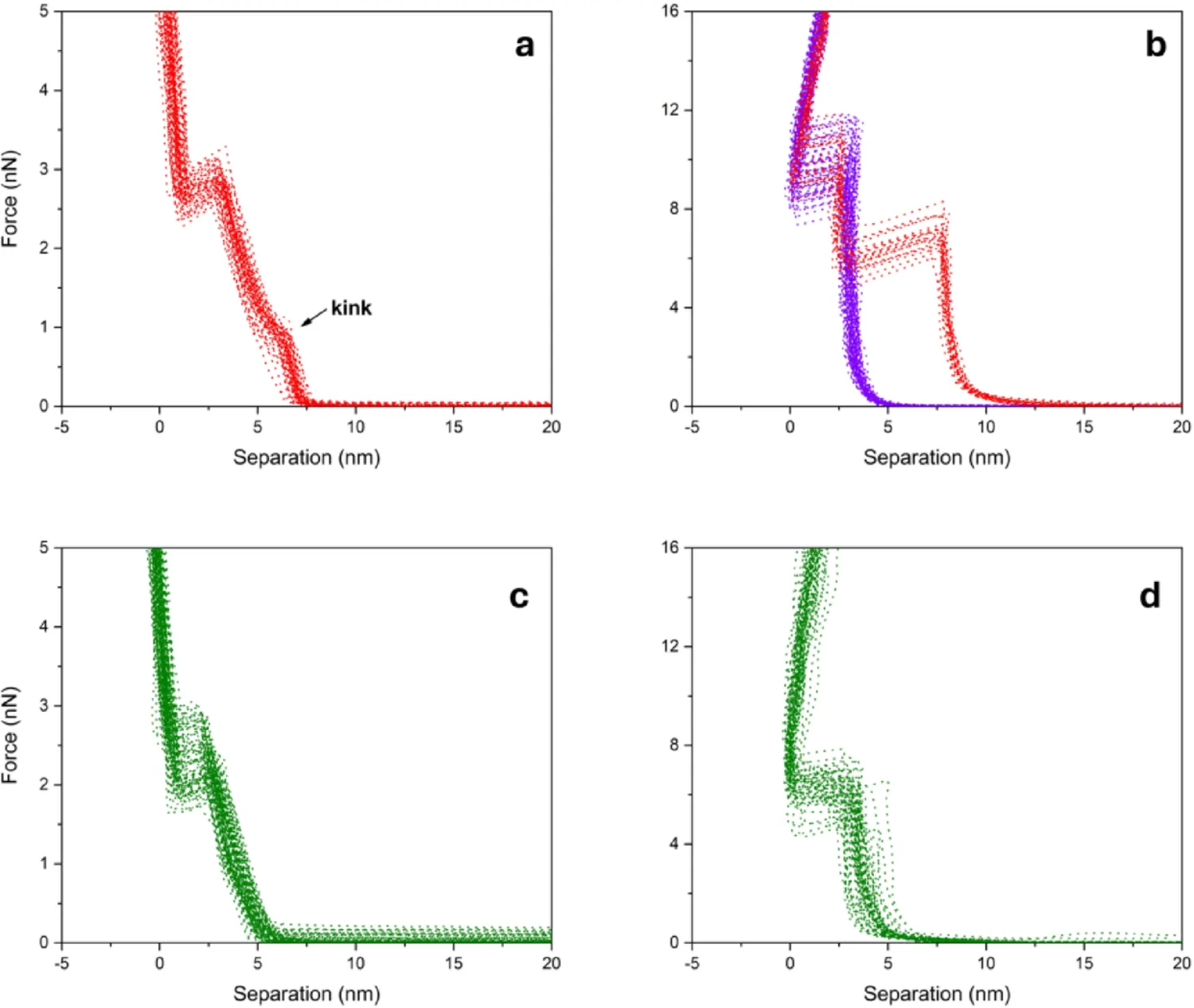
Figure 6. Force-distance (**F**-**D**) curves of lipid bilayers probed by AFM. (**a**) The DPPC/POPE (1:1) bilayer before MβCD treatment exhibits a characteristic kink (black arrow), which is indicative of leaflet asymmetry or domain formation. (**b**) The DPPC/POPE/Chol (1:1:2) bilayer exhibits two distinct breakthrough points before MβCD treatment, suggesting intrinsic asymmetry due to the presence of cholesterol-rich domains. (**c**) The DPPC/POPE (1:1) bilayer after MβCD treatment displays a symmetric rupture profile and a loss of the kink, reflecting disrupted bilayer integrity. (**d**) The DPPC/POPE/Chol (1:1:2) bilayer after 1 h of MβCD treatment shows a single, symmetric breakthrough point, which is consistent with cholesterol extraction and bilayer homogenization. At least 55 points of measurements were collected. Two distinct colors correspond to separate puncture events in the lipid bilayer, indicating the presence of layers with differing thicknesses and mechanical stiffness.

The force-distance (F–D) curves presented in Fig. 6 reflect the mechanical responses of lipid bilayers subjected to indentation by an atomic force microscope (AFM). Figure 6a shows that the DPPC/POPE (1:1) bilayer exhibits a characteristic kink at a separation of around 9–11 nm. This kink usually indicates the presence of asymmetry or phase heterogeneity within the bilayer, which is likely caused by partial lipid interdigitation or differences in leaflet packing. This first kink represents a mechanical transition before the tip fully enters the bilayer’s main body—and such transitions originate from structural or compositional differences within the membrane. As the AFM tip penetrates, it first encounters a softer or distinct domain, which cause the initial kink followed by a deeper region (main bilayer puncture). Such behavior is expected in mixed lipid systems such as DPPC/POPE, where differences in headgroup size and tail saturation can lead to non-uniform membrane properties (Alessandrini and Facci 2012; Vázquez et al. 2021).

Upon MβCD treatment (Fig. 6c), the characteristic kink observed in the F–D curves of the DPPC/POPE bilayer disappears. This indicates that structural reorganization of the membrane results in a more symmetric bilayer. Compared to the untreated bilayer (**red**,Fig. 6a), the treated bilayer (Fig. 6c, **green**) shows a slight decrease in breakthrough force which is reflecting reduced transbilayer complexity. After MβCD treatment, the breakthrough forces are distributed between approximately 1.8 and 2.9 nN.

Figure 6b shows two distinct breakthrough events at higher forces (~ 6–12 nN) for the cholesterol-containing system (DPPC/POPE/Chol, 1:1:2), suggesting the presence of a more complex and stiffer bilayer architecture. The presence of cholesterol increases the mechanical strength of the bilayer by increasing its packing density. This is consistent with the higher force requirements for breakthrough (Chen & Rand, 1997; Pan et al. 2008; Chakraborty et al.^ 2020^).

The two F-D curves (red and violet) are indicative of asymmetry or heterogeneity in leaflet composition or domain segregation within the bilayer (Vázquez et al. 2021). Additionally, the two puncture events observed in the force–displacement (F–D) experiment of the bilayer are likely caused by increased mechanical stability from the presence of cholesterol. Moreover, the distribution of cholesterol leads to decoupling between the membrane leaflets, further contributing to the observed behavior. The red curve shows two breakthrough points that commonly occur in cholesterol-rich bilayer (Busto et al. 2014; García-Arribas et al. 2015; Adhyapak et al. 2018; Khadka et al. 2021). For a single puncture phenomenon, after indenting the membrane, the tip moves through its hydrocarbon core. It then passes a thin water layer located beneath the membrane before reaching the mica substrate. The water layer thickness is around 10–20Å serving as a lubricant and helping maintain lipid molecule mobility (Castellana and Cremer 2006).

According to Khadka et al. (2021), the first observed puncture force in such membranes corresponds to the presence of both cholesterol-rich and pure cholesterol zones for two puncture (indenting) events. The second puncture force is associated with a ‘cholesterol buffer pocket’ that forms in the buffer layer above the mica surface due to the height difference between the two domains. Since pure cholesterol domains are approximately 3.4 nm thick (Jacob et al. 2001; Mason et al. 2003) and cholesterol-rich regions are around 4.5 nm thick (Plesnar et al. 2012), the difference in height likely leads to the formation of a water or buffer pocket (Khadka et al. 2021).

In the proximal region of the bilayer — the area closest to the AFM tip, where initial contact and interaction occur — this gap creates additional buffer thickness. This thickness takes the form of a cholesterol buffer pocket alongside the buffer layer found in the typical cholesterol-rich membrane zone. The buffer pocket provides additional resistance to that arising from the leaflet headgroups alone. Consequently, it acts as a barrier that prevents the AFM tip from penetrating in a single step. It is only when sufficient force is applied by the AFM tip that it breaks through this layer, resulting in the second puncture event (Khadka et al. 2021).

After MβCD treatment, shown in Fig. 6d, the DPPC/POPE/Chol (1:1:2) bilayer demonstrates a shift to a more symmetric profile, with a single, more uniform breakthrough point. This transition indicates that MβCD disrupted domain organization, resulting in increased mechanical homogeneity. The loss of distinct domains suggests that lateral lipid organization, rather than specific components, governs the bilayer’s mechanical differentiation. 

**Fig. 7:**
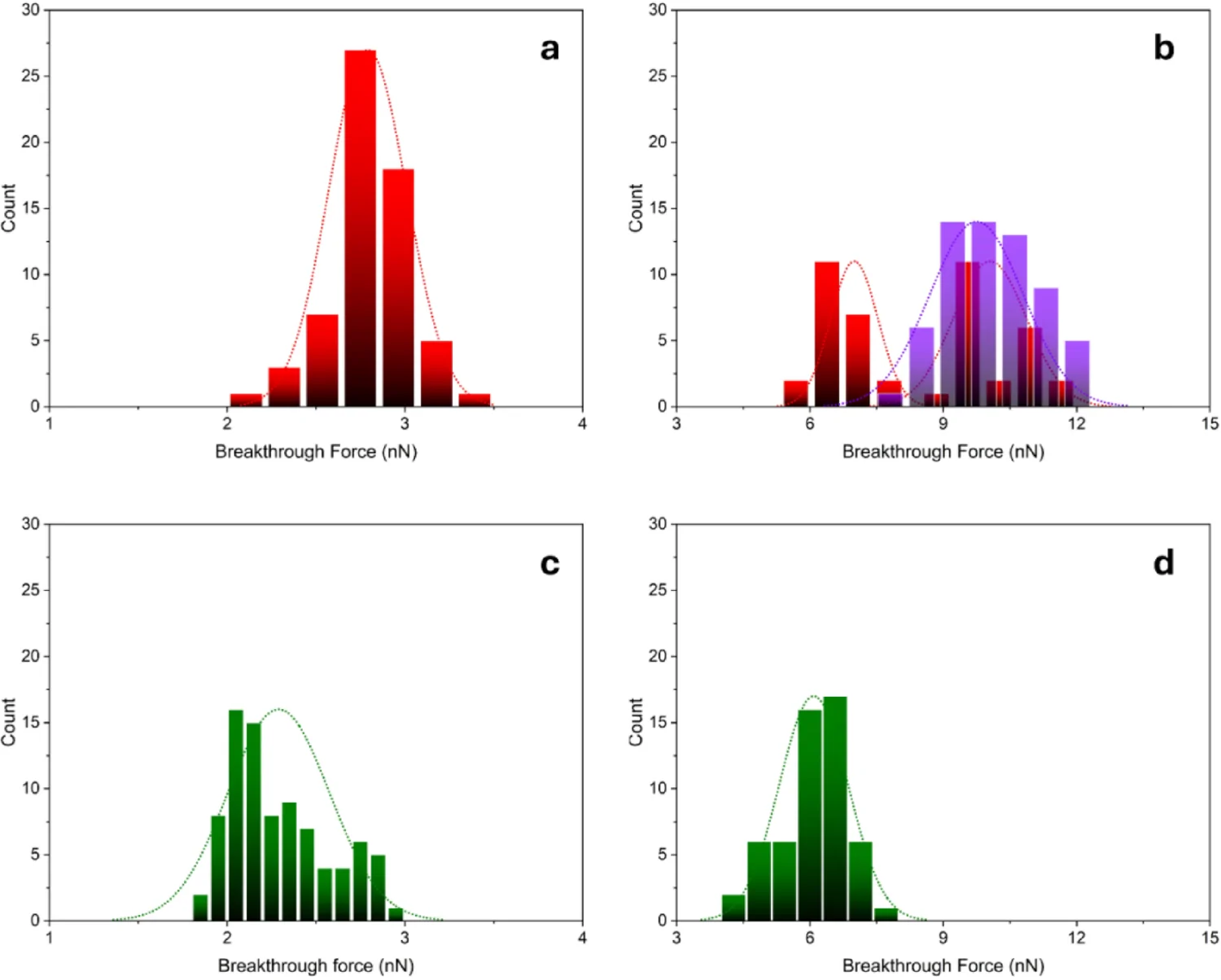
Figure 7. Histograms of breakthrough forces derived from AFM force-distance (F-D) curves for different lipid bilayer systems. (**a**) The DPPC/POPE (1:1) bilayer before MβCD treatment exhibits a narrow distribution centered around 2.7–2.9 nN (red), suggesting a relatively uniform bilayer structure. (**b**) The DPPC/POPE/Chol (1:1:2) bilayer beforep MβCD treatment displays a bimodal distribution (red and violet), representing two distinct breakthrough events. This is consistent with asymmetric bilayer organization. (**c**) After 1 h of MβCD treatment, the DPPC/POPE (1:1) bilayer shows a broadened and shifted distribution (green), suggesting bilayer symmetry. (**d**) DPPC/POPE/Chol (1:1:2) after MβCD treatment exhibits a single symmetrical distribution centered at 6 nN (green), indicating that the bilayer has been homogenized following cholesterol extraction. Each panel reflects a set of at least 55 force-distance (F-D) curves, with colored Gaussian fits corresponding to the puncture populations observed in earlier atomic force microscopy (AFM) measurements

The histograms in Fig. 7 quantitatively reflect the mechanical properties of lipid bilayers under AFM indentation, revealing the force thresholds required to puncture the bilayers. Figure 7a corresponds to the DPPC/POPE (1:1) bilayer before MβCD treatment and displays a relatively narrow Gaussian distribution centred at 2.8 nN. This sharp, unimodal peak indicates relatively uniform membrane architecture and consistent breakthrough force. Although previous F–D curves showed a subtle kink, this feature is not strongly reflected in the histogram, which represents only the puncture forces.

Figure 7b illustrates the effect of cholesterol incorporation on the mechanics of the DPPC/POPE/Chol (1:1:2) bilayer. The histogram is clearly bimodal, with red and violet distributions corresponding to two distinct populations of breakthrough forces of approximately 6.5 nN and 9.5 nN, respectively. These results support the idea that the bilayer is asymmetric, which is likely due to an uneven distribution of cholesterol across the two leaflets or the formation of cholesterol-rich and -poor domains. This is observed in case of high cholesterol (C > 20%) content as reported in literature (Adhyapak et al. 2018). These two force populations corroborate with the earlier F-D observation of two puncture points, indicating mechanically distinct regions within the membrane.

After MβCD treatment, as seen in panel (c), the DPPC/POPE (1:1) bilayer exhibits a dispersed force distribution. The green regions indicate a decrease in breakthrough forces in the post-treatment bilayer. Similarly, the DPPC/POPE/Chol (1:1:2) bilayer (Fig. 7d) becomes mechanically homogenized, with the previously bimodal distribution collapsing into a single peak around 6 nN. These changes suggest that MβCD effectively removes cholesterol and phospholipids, leading to a more uniform bilayer structure.

Overall, the cholesterol-containing SLB exhibits greater mechanical strength than the DPPC/POPE (1:1) bilayer, as evidenced by higher breakthrough forces and steeper gradients in the force–distance curves. These findings highlight cholesterol’s role in promoting bilayer asymmetry and enhancing mechanical resistance, while its removal by MβCD leading to a mechanically homogenized membrane. In the absence of cholesterol, MβCD disrupts asymmetric bilayer into domain segregation, resulting in reduced puncture forces.

We then evaluated the distribution of breakthrough force values using statistical analysis. A t-test was employed to determine the significance of the difference between the mean values of the two bilayer systems tested. The results showed p-values of less than 0.001 for both systems. These findings indicate that the mean breakthrough forces of the SLBs before and after MβCD treatment were significantly different. This suggests that MβCD can disrupt SLBs both with and without cholesterol, under the conditions of this study.

## Conclusion

This study reinforces the crucial role of cholesterol in shaping the structural and mechanical characteristics of lipid bilayers. In the absence of cholesterol, DPPC/POPE bilayers formed symmetrically after MβCD treatment and displayed moderate mechanical strength. Incorporation of cholesterol into the DPPC/POPE/Chol system introduced pronounced asymmetry and elevated bilayer stiffness, as reflected by the presence of dual breakthrough events in AFM force–distance measurements. Upon treatment with MβCD, cholesterol was effectively extracted, restoring bilayer symmetry and reducing mechanical variability. These transitions were corroborated by DLS, zeta potential, and QCM analyses, with the latter revealing a temperature-dependent profile for cholesterol removal. Overall, this work sheds light on the dynamic behavior of cholesterol-rich membranes and underscores the value of AFM and QCM as complementary tools for probing lipid bilayer remodelling.

## Data Availability

All data supporting the findings of this study are available upon request.
